# Interstate Reciprocity in Medical Licenses

**Published:** 1906-09

**Authors:** 


A Monthly Review of Medicine and Surgery.

*EDITOR:*

WILLIAM WARREN POTTER, M. D.

All communications, whether of a literary or business nature, books for review and exchanges, should be addressed to the editor 284 FRANKLIN ST., BUFFALO, N. Y.

VOL. LXII. SEPTEMBER, 1906. No. 2

### Interstate Reciprocity in Medical Licenses.

A REVIVAL of interest in reference to state exchange of licensure courtesies seems to be pervading the medical profession of late. The subject is being discussed in medical journals and medical societies and, as it seems to us, altogether too much importance has been accorded this interlocutory question relating to state control of medical education.

It would be desirable on some accounts if this republic were made up of a single commonwealth, instead of being composed of forty-six separate states and a few territories. It would simplify many questions that now arise to perplex lawyers, courts and individuals. The divorce laws, for example, would be unified, the criminal code would apply in a uniform manner to every citizen of the republic, and many other matters of law and administration would be reduced to a simple process.

Reciprocity is of course desirable but it is not essential to the successful working of our medical laws toward the final goal of uniformity in standards. We have pointed out on more than one occasion that the surest route to reciprocity is through uniform methods of admission to our medical colleges, equal standards of teaching, and length of terms of study. It would seem out of place for medical colleges to demand reciprocity while there is still so much that is unequal in their own methods.

We are quite prepared to understand how some of the smaller colleges feel disturbed at state supervision or control. They have been accustomed so long to regulate their own affairs that restraint chafes when applied or adjusted by a power that cannot be resisted. So many items of prime importance require attention that secondary questions, like reciprocity, may with propriety be allowed to stand in abeyance for the present.

We have been led to return to this subject because of a paper by Dr. James A. Egan, secretary of the Illinois state board of health, read before the Illinois State Medical Society in May,

1906, and recently distributed as a reprint, the JOURNAL having been complimented with a copy. Dr. Egan's knowledge of the subject is comprehensive and intimate and he discusses it with great perspicacity. He takes up the question from legal as well as from medical viewpoints, and we advise all interested to obtain a copy of this interesting reprint.

In a supplement to his paper he pays his compliments to Dr. William J. Mayo, president of the American Medical Association, quoting from his presidential address at Boston, as follows:

> Another question of great importance is that of reciprocity in medical license. The conditions are now well-nigh intolerable and restrain the individual freedom guaranteed by the constitution. The boundaries between the states are imaginary lines; yet a physician on one side of the border cannot relieve human suffering on the opposite side without becoming amenable to the law or subjecting himself to vexatious examinations which he has already successfully passed in his own state. This must be met and speedily by agreement between examining boards as to the minimum of requirements.

Dr. Egan then comments in detail on this statement of President Mayo in the following language:

In connection with Dr. Mayo's remarks the editor of the Journal of the Association, in the issue of June 9th, says: "there are few men who will not agree with him in what he says in regard to state licensure and reciprocity."

It may be that there are many who will agree with Dr. Mayo that the conditions now existing "are well-nigh intolerable," but with all respect to President Mayo and the editor of the Journal of the Association, there are but few who have given the question of national and state control of the regulation of the practice of medicine proper consideration who can agree that the conditions set forth by Dr. Mayo actually exist.

In the logical consideration of a problem, we must deal with facts, not with fancies. In an attempt to solve a problem we must, if we are to attain success, deal with the finite, not the infinite; the possible, not the impossible; with practice, not with theory.

The right to practise medicine is not a part of the individual freedom guaranteed by the Constitution. As has been aptly stated "the individual states were in no way divested of their sovereignty by the adoption of the United States Constitution, except so far as that instrument shows an express surrender of their sovereignty." The regulation of the practice of medicine comes within the police power of a state, a power which the states have retained unimpaired since 1789.

The boundaries between the states are imaginary lines, in but imagination. In reality the state's boundary lines are as stable and definite as the national boundary lines. State lines existed prior to the national lines. In the original establishment of the United States, the state lines were the definite and well defined lines, while the federal lines have remained expansive to be adapted to the extension of the states constituting the Union. The line between Illinois and Indiana, where no geographical line of demarcation is to be found, is quite as definite and exact as the line between the United States and Canada, or that between Austria-Hungary and the Russian Empire.

The necessity for a physician on one side of the border of a state relieving human suffering on the opposite side, is not obvious except in cases of dire distress, where an emergency exists. Under these circumstances a border line physician can practise without let or hindrance, and no state board will interfere with him. But there is seldom an emergency, seldom a case of dire distress, which cannot be given attention by the physicians of the state in which it occurs. Nearly every state has its quota of capable and qualified physicians.

But it has been said that a citizen of one state taken ill in another state cannot be attended by his family physician unless the latter be licensed to practise in the state in which the patient lives. I deem it sufficient to call for statistics of prosecutions instituted in the various states on account of practice of this character. I refer now to practice in an individual case, by the bona fide family physician. I do not refer to the practice of the itinerant. I do not refer to the practice of the tourist who is prone to consider himself the family physician of all persons,—and their friends, relatives and acquaintances,—coming from his native state. I do not refer to the practice of the physician who, when summoned to another state to treat his patient, treats all who apply to him. But even now the laws of nearly forty states permit the practice of physicians from other states in consultation.

It would be exceedingly difficult for any state, which must retain the controlling power in medical licensure until the constitution of the United States is radically changed, to draw a line which would limit the invasion of physicians from other states if the natural state boundary lines were ignored. The state line must be the logical and only practical limitation for the practice of the physicians of each individual state.

As to the limitations placed upon practitioners of the various states, the statements of Dr. Mayo are misleading. The restrictions upon those who have already successfully passed examinations in their own states, are gradually disappearing under the present systems of reciprocity. Few physicians who have successfully passed examinations in their own states are required to pass examinations in other states. Thirty-seven states are now empowered to reciprocate; of these twenty-six do reciprocate. But it must be borne in mind that the percentage of physicians who have passed examinations of any kind in their own states is small.

It may be added further, in commenting upon the brief extracts quoted from Dr. Mayo's presidential address, that, among the majority of boards which have found reciprocal relations consistent with the laws under which they operate, a definite understanding has been reached in regard to the minimum requirements of medical education.

Dr. Mayo is a learned man and careful of his statements, but in this instance he seems to have spoken hastily, basing his remarks on the ill-considered journalistic material which is afloat, rather than upon a careful study of the situation on his own part. Dr. Egan, however, has made complete answer to the distinguished president and it is hardly worth while for us to pursue the matter in further detail.

